# Risk factors for *Echinococcus* coproantigen positivity in dogs from the Alay valley, Kyrgyzstan

**DOI:** 10.1017/S0022149X15000590

**Published:** 2015-11

**Authors:** A. Mastin, F. van Kesteren, P.R. Torgerson, I. Ziadinov, B. Mytynova, M.T. Rogan, T. Tursunov, P.S. Craig

**Affiliations:** 1 Cestode Zoonoses Research Group, School of Environment and Life Sciences, University of Salford, Salford, M5 4WT, UK; 2 Department of Psychology, University of Michigan, 1012 East Hall, 530 Church Street, Ann Arbor, MI, 48109-1043, USA; 3 Section of Epidemiology, Vetsuisse Faculty, University of Zürich, Switzerland; 4 Parasitology group, Kyrgyz Veterinary Research Institute, Togolok Moldo 60, Bishkek, Kyrgyzstan

## Abstract

Echinococcosis, caused by the zoonotic cestodes *Echinococcus granulosus* (sensu lato) and *Echinococcus multilocularis,* is highly endemic in the Central Asian Republic of Kyrgyzstan, and is being identified increasingly as a public health problem, especially amongst pastoral communities. As domestic dogs are considered to be the main source of human infection, the identification of potential transmission pathways is of relevance when considering implementing an echinococcosis control scheme. The current report describes the results of an analytical study of canine *Echinococcus* coproantigen enzyme-linked immunosorbent assay (ELISA) prevalence in the Alay valley of southern Kyrgyzstan prior to the commencement of regular praziquantel dosing of dogs. A logistic regression model using a form of Bayes modal estimation was used to identify possible risk factors for coproantigen positivity, and the output was interpreted in a Bayesian context (posterior distributions of the coefficients of interest). The study found that sheepdogs had lower odds of coproantigen positivity, as did dogs in households with donkeys, where owners had knowledge of echinococcosis, and households which engaged in home slaughtering. Surprisingly, there was no evidence of an association between free roaming or previous praziquantel dosing and coproantigen positivity, as has been found in previous studies. Possible reasons for these findings are discussed in the context of the epidemiology of echinococcosis and potential intervention approaches.

## Introduction

Human echinococcosis, caused by infection with the metacestode stage of cestodes of the genus *Echinococcus*, is an important public health concern in various parts of the world. Due to the parasite's complex life cycle and long period between infection and clinical signs in human hosts, accurate investigation of risk factors for human infection can be challenging. However, where domestic dogs act as a definitive host (most areas of *Echinococcus* *granulosus* endemicity and some areas of *E. multilocularis* endemicity (Craig *et al.*, 1992)), identification of risk factors for canine infection can provide useful information on potential human risk, and can be useful for designing and monitoring *Echinococcus* control schemes based on treatment of infection in dogs. Although *Echinococcus* spp. infection in dogs is asymptomatic, a number of diagnostic tools are available for diagnosis of current infection. Detection of coproantigens is of particular use and has been advised as the mainstay of surveillance of echinococcosis in endemic areas by the World Health Organization (WHO), World Organization for Animal Health (OIE) and Pan American Health Organization (PAHO) (WHO/OIE, 2001; Morel *et al.*, 2013).

As the transmission cycle of *Echinococcus* spp. may vary between locations, it is useful to identify risk factors specific to the particular transmission system in question. It is also useful to evaluate commonly identified risk factors from the wide range of studies that have been conducted worldwide (Otero-Abad & Torgerson, 2013). Coproantigen test results are often used to approximate canine infection status with *Echinococcus* spp. – classifying samples as coproantigen ‘negative’ or ‘positive’, according to their enzyme-linked immunosorbent assay (ELISA) optical density (OD) value in relation to a defined cut-off value. This will lead to some misclassification, which has been addressed in some studies by combining the results of purgation and coproPCR (Ziadinov *et al.*, 2008). Potential risk factors for infection can be classified according to a number of general transmission processes: factors associated with access to infected material (infected offal, in the case of *E.* *granulosus*, or small mammal intermediate hosts in the case of *E.* *multilocularis*); factors associated with variability in infection after ingestion of infectious material; and factors associated with removal of infection, such as a history of anthelmintic treatment. The most commonly identified risk factors are those relating to access to infected material, including access to offal or infected rodents (Bchir *et al.*, 1987; Parada *et al.*, 1995; Moro *et al.*, 1999; Y.H. Wang *et al.*, 2001; Shaikenov *et al.*, 2003; Budke *et al.*, 2005; Buishi *et al.*, 2005b, 2006; Q. Wang *et al.*, 2007, 2010; Dyachenko *et al.*, 2008; Guzel *et al.*, 2008; Huang *et al.*, 2008; Ziadinov *et al.*, 2008; Antolová *et al.*, 2009; Acosta-Jamett *et al.*, 2010; Mastin *et al.*, 2011; Reyes *et al.*, 2012).

Risk factors have also been identified at the dog level: in particular, dog type and age. Working dogs such as sheepdogs and farm dogs (Moro *et al.*, 1999; Shaikenov *et al.*, 2003; Buishi *et al.*, 2005a) have been found repeatedly to have higher odds of infection with *E.* *granulosus*, which likely relates to increased availability of, and access to, potentially infectious material. Younger dogs have also been found repeatedly to have higher odds of infection than older dogs (Sharifi & Zia-Ali, 1996; Buishi *et al.*, 2005a, 2006; Acosta-Jamett *et al.*, 2010; Inangolet *et al.*, 2010). This could result from differences in feeding behaviour between younger and older dogs. Alternatively, modelling approaches have suggested that this could represent immunity acquired in young dogs preventing parasite acquisition as the dog ages, which may be more apparent for *E.* *granulosus* than for *E.* *multilocularis* infection (Torgerson *et al.*, 2003; Torgerson, 2006). Finally, a lack of recent anthelmintic dosing has been commonly identified as a risk factor for canid infection (Parada *et al.*, 1995; Buishi *et al.*, 2005a; Huang *et al.*, 2008; Acosta-Jamett *et al.*, 2010).

It should be noted here that studies of risk factors for infection of domestic dogs (rather than foxes) with *E.* *multilocularis* are relatively uncommon. Domestic dog infection with *E.* *multilocularis* is most commonly only identified in particular pastoral communities, such as Tibetan communities in China or Kyrgyz communities in Kyrgyzstan (Budke *et al.*, 2005; Q. Wang *et al.*, 2007, 2010; Ziadinov *et al.*, 2008). However, recent work has also identified infection in domestic dogs (albeit often at very low levels) in central and eastern Europe (Deplazes *et al.*, 1999; Dyachenko *et al.*, 2008; Antolová *et al.*, 2009), and so evaluation of risk factors for *E.* *multilocularis* in domestic dogs may become increasingly common.

The current study investigates risk factors for canine *Echinococcus* spp. coproantigen positivity in the Alay valley in the Osh Oblast of Kyrgyzstan – an area of known high endemicity of human alveolar echinococcosis (Usubalieva *et al.*, 2013). A combination of Bayesian and frequentist strategies were utilized in order to identify and describe these risk factors.

## Materials and methods

### Study sites

In May 2012, four communities in the Alay valley of southern Kyrgyzstan were visited (Sary Mogol (39.68°N, 72.89°E), Taldu Suu (39.70°N, 72.98°E), Kashka Suu (39.64°N, 72.67°E) and Kara Kavak (39.66°N, 72.72°E)), prior to the commencement of a praziquantel-based pilot intervention for canine echinococcosis. A more detailed description of the study site can be found elsewhere (van Kesteren *et al.*, 2013). All occupied households in Sary Mogol, Taldu Suu and Kara Kavak, and a random selection of approximately 25% of the households in Kashka Suu were visited. For each household visited, a questionnaire was administered relating to details such as general demographics (age, sex, occupation of interviewee), dog ownership (number of dogs currently owned, management of these dogs), dog demographics (dog age, dog sex, dog weight) and perception of echinococcosis (recent administration of praziquantel to dogs, understanding of source of human echinococcosis). However, not all questions were answered by all interviewees. Of 692 households registered, a total of 329 individuals reported owning dogs, and a total of 388 dogs in total were registered. All questionnaire data were entered into Microsoft^®^ Access.

### Collection and examination of faecal samples

Faecal samples were taken from the available owned dogs. Samples were collected rectally when possible, but otherwise were taken from the ground around the homestead, with attempts made to match individual samples to individual dogs. Each sample was divided and each part stored in either saline buffer (phosphate-buffered saline (PBS) with Tween) or ethanol before transportation to the University of Salford, England, where they were stored at − 80°C for a minimum of 5 days prior to testing (WHO/OIE, 2001). A total of 318 faecal samples were available. These were tested using a standardized sandwich coproantigen ELISA (Allan *et al.*, 1992), with modifications in that the capture and conjugate antibodies were raised from two different hyperimmune rabbit sera (van Kesteren *et al.*, 2015). All samples were tested using the same reagents in the same ‘batch’ period of no more than 4 days, with each sample tested in duplicate in adjacent wells. For controls, a parasitologically defined faecal panel of necropsy dog samples was available as described in van Kesteren *et al.* (2015).

### Data analysis

Initial data processing was conducted using Microsoft^®^ Access 2010, and all further data processing and analysis was conducted using R version 3.1.1 (R Development Core Team, 2014). The difference in coproantigen ELISA OD between the two duplicates for each sample was calculated and the Studentized residuals of an intercept-only linear regression were inspected for outliers. A Bonferroni-corrected *t*-test was conducted using the ‘outlierTest’ function in the ‘car’ package for R (Fox & Weisberg, 2011), and any samples that gave a *P* value of 0.05 or less were removed from further analysis as possible failures of replication. Of the 318 faecal samples, 23 could not be matched to an individual questionnaire (due to illegible or damaged sample labels), but were retained in the model as the village of origin was known. Receiver-operator characteristic (ROC) curve analysis (Zweig & Campbell, 1993; Greiner, Pfeiffer & Smith, 2000) was used on a panel of parasitologically defined dog faecal samples taken from Xinjiang province in China (van Kesteren *et al.*, 2015). The Youden index approach, i.e. maximization of both test sensitivity and specificity (Youden, 1950) was used to determine the optimal cut-off point. The resultant cut-off point (OD 0.07635) gave an estimated test sensitivity of 96% and specificity of 83%, based upon the panel evaluated.

Prior to analysis, the number of variables with missing data was assessed. All variables with more than 250 missing data points were removed from further analysis, as were those categorical variables with fewer than five outcomes in any single category. This process left a total of 41 variables to be investigated (table 1).

**Table 1:** Variables considered in the risk factor modelling process; livestock ownership was evaluated using both a dichotomous variable (presence/absence) and a continuous variable (number of animals owned).

Variable type	Variables
Village	Village
Animal ownership	Current number of dogs
	Number of dogs owned in past 10 years
	Sheep
	Goats
	Cattle
	Horses
	Yaks
	Donkeys
Dog demographics	Age
	Size (small/medium/large)
	Weight
	Sex
	Used for hunting
	Guard dog
	Pet dog
	Sheepdog
Dog management/behaviour	Wormed in past 6 months
	Percentage of time spent free roaming
	Known to eat rodents
	Fed meat
	Fed offal
	Chained at all
	Handled by adults from the household
	Handled by children from the household
	Handled by friends of the family
	Not handled
	Visited pasture previous year
	Will visit pasture this year
Animal slaughter	Home slaughter, own
	Home slaughter, others
	Organs thrown away
	Organs given to dogs
	Organs buried
Perceived source of human echinococcosis	Dogs
Cats
	Livestock
	Unknown

Initial analysis utilized simple non-parametric univariable methods (Fisher's exact test, chi-square test or Mann–Whitney *U*-test) to identify those variables with some evidence of association with coproantigen status (using a *P* value of less than 0.3 to suggest some association). Any collinear parameters identified at this stage were reduced to one parameter based on the *P* value obtained. Twelve variables were selected for inclusion in the preliminary regression model.

All variables identified in the previous stage were added to a Bayesian logistic regression model, using the ‘bayesglm’ procedure in the ‘arm’ package (Gay *et al.*, 2008; Gay & Su, 2014) for R. This procedure incorporates ‘vague’ priors based upon Cauchy distributions into the regression model using data augmentation techniques (Cole *et al.*, 2012). The log posterior density [log *p*(β, σ|*y*)] is maximized using an iterative process combining weighted least squares (Nelder & Wedderburn, 1972; Fox, 2008) and an expectation-maximization (EM) algorithm (Dempster *et al.*, 1977) in order to obtain parameter estimates. In line with the output of the ‘glm’ procedure upon which it is based (R Development Core Team, 2014), coefficient estimates are provided as point estimates along with their standard errors.

Model selection was based upon a manual stepwise removal process according to their Wald test *P* values, and a likelihood ratio test was used to identify possible contribution to the model (with a *P* value of 0.1 or less suggesting some contribution). Confounding was assessed by monitoring coefficients of other variables before and after variable removal, with a change of 30% or more suggestive of possible confounding. Where coefficients were less than 0.001 in magnitude, an absolute change in the magnitude of the coefficient of 0.001 or more was used to indicate a potentially confounding effect. Following this process, all plausible interactions between the remaining variables, and any quadratic and cubic trends in any continuous variables, were assessed using a likelihood ratio test, with a *P* value of 0.05 or less suggestive of a significant effect. Model diagnostics were conducted using residual plots and influence plots, and observations removed as appropriate. Variables were then removed sequentially from the final model, using a likelihood ratio test of 0.05 or less to suggest model contribution. The fit of the final model was assessed using a likelihood ratio goodness-of-fit test.

Posterior simulation using the ‘sim’ procedure in the ‘arm’ package was used with 10,000 iterations in order to approximate Bayesian posterior estimates of coefficients in the final model. The simulation output was exponentiated in order to obtain estimates of the posterior distribution of odds ratio estimates. These distributions were then summarized using the mode (as estimated from the kernel density, according to Parzen (1962), using the ‘modeest’ package in R (Poncet, 2012)) and the highest density intervals (HDI) (using the ‘HPDinterval’ procedure in the ‘coda’ package (Plummer *et al.*, 2006)).

## Results

**Fig. 1:**
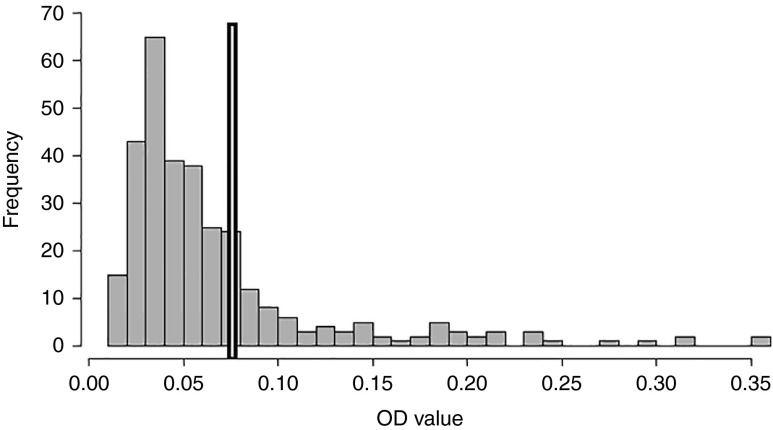
The distribution of coproELISA OD values for all 318 dog faecal samples tested; cut-off for positivity at 0.07635 (bold vertical bar).

A total of 78 out of 318 canine faecal samples (25%) tested coproantigen positive using the cut-off as calculated from ROC curves (table 2). The distribution of OD values from these samples showed a clear right skew, as shown in fig. 1. There was no evidence of a difference in coproantigen prevalences between villages (*P*= 0.5) (table 2).

**Table 2:** Numbers of canine faecal samples analysed from the four study villages in the Alay valley together with point estimates of the *Echinococcus* coproprevalence (%). Confidence intervals are not shown as the data were collected by census from all villages with the exception of Kashka Suu.

Village	Proportion of total samples	Number of samples	Coproprevalence (%)
Sary Mogol	0.49	155	27
Taldu Suu	0.27	86	19
Kara Kabak	0.13	42	24
Kashka Suu	0.11	35	29

Ten categorical variables were found to be associated with coproantigen status at the end of the first stage of analysis (*P* value < 0.3). None of the continuous variables were found to be associated. Of the categorical variables, two variables relating to donkey ownership were identified as associated with coproantigen status: one based upon a dichotomous classification of donkey ownership, and one where donkey ownership was categorized according to the number of donkeys owned. As the latter variable was found to have a higher *P* value than the former, this was removed from further analysis. The variables found to be associated with coproantigen status in the first stage of analysis are shown in table 3, along with the associated *P* values.

**Table 3:** *Echinococcus* coproprevalences (%) in dogs relative to variables identified during univariable analysis; *N*=295 respondents.

Variable	Negative respondents	Positive respondents	Coproprevalence amongst dogs of negative respondents (%)	Coproprevalence amongst dogs of positive respondents (%)	*P* value (all < 0.3)
Hunting dog	227	68	26	18	0.21
Home slaughter practised	14	281	7	25	0.23
Organs thrown away	200	95	22	28	0.29
Dogs perceived source of hydatid	283	12	25	0	0.10
Cats perceived source of hydatid	288	7	24	0	0.29
Sheepdog	251	44	26	11	0.05
Dog handled by adults	139	156	21	27	0.28
Owns donkeys	165	130	29	18	0.03

At the end of the second stage of analysis, home slaughter, knowledge of hydatid source in dog, sheepdog ownership and donkey ownership variables were found to be associated with coproantigen status, with no evidence of interaction between variables. In the final stage of model selection, likelihood ratio tests found all four remaining variables to be significant at *P* <  0.05. The likelihood ratio goodness-of-fit test gave a *P* value of 0.27, suggesting a reasonable model fit. The exponents of the simulated posterior estimates (which describe the change in the log odds of infection associated with each variable) were calculated and the resultant distributions summarized in order to estimate the odds ratios of the effect of the different variables on coproantigen positivity (table 4).

**Table 4:** Odds ratios of the variables included in the final regression model.

Variable	Odds ratio (mode)	95% highest density interval
Home slaughter practised	2.04	0.18–18.46
Dogs perceived source of human hydatid disease	0.03	0.0005–0.95
Sheepdog	0.27	0.09–0.77
Owns donkeys	0.46	0.24–0.77

## Discussion

Using the *Echinococcus* ELISA coproantigen cut-off described here, the overall canine coproantigen prevalence in the study area was estimated as 25%. Due to the limitations in the coproantigen test, especially in the case of low worm burdens (Allan & Craig, 2006), the true prevalence is likely to differ from the coproantigen prevalence, and it is for this reason that coproantigen prevalence estimates are not presented to any higher precision here. The aim of the current study was not to estimate the prevalence of infection, but to identify possible risk factors for infection. Four risk factors were found to be associated with reduced odds of coproantigen positivity: ownership of donkeys, description of the dog as a sheepdog, knowledge that dogs are a source of human echinococcosis and a lack of home slaughtering in the household.

The logistic regression modelling framework used for the current study utilized a combination of frequentist and Bayesian methodologies: a Bayesian prior was incorporated into the model in order to ensure model identifiability even when data were sparse, as was the case with home slaughter and owner knowledge of risk factors for human echinococcosis. Model selection was based on frequentist interpretation of coefficient estimates, but as the model likelihoods incorporated the prior, the likelihood ratio test used for model comparison could be considered partly Bayesian. Initial interpretation of the final model used both approaches, but the final conclusions were based upon Bayesian posterior estimates. The distinction between these approaches is of importance in terms of the communication of model selection and the final conclusions. The use of well-known frequentist strategies, such as likelihood ratio testing, ensures that the model selection process can be understood by people not familiar with Bayesian methods, but the final interpretation of the model output in a Bayesian setting makes the model output conceptually easier to understand.

Donkey ownership was found to be associated with reduced odds of owned dog coproantigen positivity. This is an unexpected finding, and has not been reported in any previous studies. Donkey ownership may reflect a socio-economic factor: for example, people with donkeys may have more disposable income than those without, which could affect the risk of canine infection as dogs from higher-income households may be better fed and less likely to scavenge. Alternatively, donkey ownership may relate to spatial factors. Donkeys were commonly used for water transportation, and therefore households with donkeys may tend to be located further from a water source than those without donkeys. Proximity to water has been suggested repeatedly to be a factor of importance in the sylvatic *E.* *multilocularis* transmission cycle. A study of vulpine infection with *E.* *multilocularis* found that infected foxes tended to be located near water sources (Staubach *et al.*, 2001), which may be suggestive of differences in suitable intermediate host habitats or may indicate spatial aggregation of infection in intermediate hosts due to prolonged egg survival in water (Hansen *et al.*, 2003, 2004). Also, a study of *E. granulosus* infection of dogs living around eight abattoirs in Lima, Peru, found that dogs from abattoirs located close to the river were more likely to be infected with *E.* *granulosus* (Reyes *et al.*, 2012), which was suggested to potentially result from infected offal being discarded into the river. Further work to investigate spatial patterns of coproantigen positivity within the communities would be interesting – with a particular focus on *E.* *multilocularis* infection.

Sheepdogs were found to have lower odds of coproantigen positivity than other dogs. This is contrary to previous studies, which have routinely identified sheepdogs as having a higher probability of coproantigen positivity or infection than non-sheepdogs (Moro *et al.*, 1999; Buishi *et al.*, 2005a). Similarly, farm dogs have also been identified as having a higher probability of infection than village dogs in Kazakhstan (Shaikenov *et al.*, 2003; Torgerson *et al.*, 2003). In the study area, there was a clear distinction between ‘sheepdogs’ and ‘pet’ or ‘guard’ dogs (with the latter two classifications apparently being used interchangeably) (van Kesteren *et al.*, 2013). It is likely that sheepdogs were used for herding and guarding livestock during their seasonal and daily movements to pasture, whereas pet and guard dogs likely represent those dogs that remained in the village. In studies where sheepdogs were found to have higher odds of infection, this is usually considered to be due to potential access to infected offal, and therefore exposure to *E.* *granulosus*. However, *E.* *multilocularis* infection may be of particular importance in the current study area, for which contact with livestock would not be expected to be of importance. Therefore, it is possible that this association with dog type represents a spatial risk factor, with dogs based mostly within the village having a higher probability of infection than those sheepdogs that spend more time outside the village.

Both knowledge that dogs were a risk factor for human echinococcosis and lack of home slaughtering were found to be associated with a reduced probability of canine infection when assessed using the likelihood ratio test. However, the confidence intervals of the unadjusted model coefficients crossed the threshold of zero in both cases. In the case of the home slaughtering variable, this ‘non-significant’ effect persisted in the final HDI estimates (table 4). These issues are likely the result of a scarcity of positive or negative responses for these variables (table 3), and as these associations have been reported previously in the literature, they will be discussed further here as potential risk factors.

In previous studies, knowledge of cystic echinococcosis has been identified to be a significant risk factor for canine coproantigen status (Buishi *et al.*, 2005a; Huang *et al.*, 2008). Although no explicit distinction was made between cystic echinococcosis and alveolar echinococcosis in the question asked in the current study, it is likely that people with knowledge of echinococcosis are less likely to engage in practices such as feeding of infected offal which could facilitate transmission to dogs. This finding therefore may demonstrate some potential benefits of education campaigns as an adjunct to an echinococcosis control scheme.

Home slaughter has been found, in previous studies, to be positively associated with coproantigen positivity (Buishi *et al.*, 2005b; Acosta-Jamett *et al.*, 2010, 2014), and although it is likely that almost all households slaughtered animals at some point, this association is plausible. As home slaughter likely increases the risk of feeding unwanted infected offal to dogs, this association would be expected to represent *E.* *granulosus* rather than *E.* *multilocularis* infection.

The lack of any identified association between reported dog dosing history and current status in the current study may result from the fact that information on dosing history was aggregated over a period of 6 months. Since praziquantel has no residual effect after administration, dosed dogs can become re-infected immediately after dosing. Recall bias amongst owners is also likely to be present: people who have not dosed their dogs recently may report they have, and people who have dosed recently may report that they have not, which would tend to reduce any coefficient estimates towards zero. Free roaming, which is probably the most commonly identified risk factor for echinococcosis in dogs (Parada *et al.*, 1995; Budke *et al.*, 2005; Buishi *et al.*, 2005b, 2006; Guzel *et al.*, 2008; Huang *et al.*, 2008; Ziadinov *et al.*, 2008; Antolová *et al.*, 2009; Mastin *et al.*, 2011), was also not found to be associated with test status. In the Alay valley, most dogs were free to roam throughout the village, with only 28/288 dogs (10%) reported to be chained at all, and therefore a similar lack of power to that described above would be expected for this variable. However, these results are suggestive that even chained dogs are gaining access to infected material, possibly through purposeful feeding of infected offal or resulting from occasional release from restraint.

One issue with any risk factor study based on identification of ‘significant’ risk factors from a large number of possible variables is that as the number of variables considered is increased, the probability of type I errors (i.e. finding a ‘significant’ association when this is not truly the case) also increases. In total, 41 variables were assessed in the current study, meaning that with an alpha error of 0.05, approximately two associations would be expected to be identified as ‘significant’ due to random variation alone. Model selection and evaluation strategies based upon information theoretic measures may reduce this problem, and would be a useful avenue for further exploration (Burnham & Anderson, 2002). Three other major considerations are particular to this study, and should be considered when interpreting the conclusions. These are the limitations in coproantigen test sensitivity and specificity, the lack of any differentiation between *E.* *granulosus* (sensu lato) and *E.* *multilocularis*, and the fact that relatively few faecal samples were matched conclusively to individual dogs.

As alluded to above, most studies of echinococcosis based upon coproantigen data classify all samples as ‘negative’ or ‘positive’ based upon a single ELISA optical density (OD) cut-off. This strategy will generally result in some misclassification: in particular, in the case of animals with low *Echinococcus* burdens (i.e. imperfect sensitivity), and animals infected with other taeniid cestodes (i.e. imperfect specificity). Therefore, estimates of the coproantigen prevalence are likely to differ from the true prevalence of infection. While this will tend to affect the accuracy of any prevalence estimates, it may be less of an issue in the case of analytical studies, where the intention is to identify risk factors for infection. Despite this, further work is planned to reduce the reliance on coproantigen status and instead attempt to model the true infection status. This has been achieved in recent studies by explicitly accounting for diagnostic test limitations (Ziadinov *et al.*, 2008), or by avoiding the dichotomization of ELISA results completely (Choi *et al.*, 2006).

The major limitation in the current study is the lack of *Echinococcus* species discrimination. Previous work has shown that both *E.* *granulosus* sensu lato (*E.* *granulosus* G1 and *Echinococcus canadensis* G6) and *E.* *multilocularis* are present in dogs in the Alay valley (van Kesteren *et al.*, 2013), although the human health problem, to date, appears to be due mainly to *E.* *multilocularis* (Usubalieva *et al.*, 2013). While all faecal samples in the current study were collected and tested for faecal *Echinococcus* DNA, these results were not included here due to the difficulties in combining these results with the coproantigen ELISA results in a useful way. Further work will be undertaken to investigate risk factors for infection as identified by polymerase chain reaction (PCR), and it is hoped that methods of combining results obtained from these different testing methodologies, as has been achieved in other studies (Ziadinov *et al.*, 2008; Hartnack *et al.*, 2013), will be developed for co-endemic situations in due course. Development of coproantigen ELISA tests that are specific for a variety of different species and strains of *Echinococcus* (WHO/OIE, 2001), or the development of alternative tests that would allow species discrimination in a surveillance setting, would be of great use. Examples of the latter are single-tube, isothermal DNA amplification techniques, such as loop-mediated isothermal amplification (LAMP) – which has already been developed for *E.* *granulosus* G1 and *E.* *multilocularis* (Salant *et al.*, 2012; Ni *et al.*, 2014a, b) – or recombinase DNA polymerase amplification (RPA) (Piepenburg *et al.*, 2006).

Finally, despite efforts to sample dogs per rectum whenever possible, most of the samples were collected from the ground around the household, and therefore cannot be definitively matched to individual dogs (or even individual households), due to the free-roaming behaviour of the dogs. Attempts were always made to involve the owners in order to identify faeces passed by the dog in question, but this was not always possible. Therefore, it is highly likely that some of the samples analysed in the current study are not from the dogs for which questionnaire data were collected. As the correct identification of an individual dog is unlikely to be associated with the coproantigen status of that dog, there is no reason to believe that this will result in directional bias, but this sampling strategy would be expected to reduce the study power. This problem of identifying samples from individual dogs would also be expected to be a problem in *Echinococcus* surveillance schemes, where ground samples are collected from free-roaming dogs. Further work to determine an optimal strategy to deal with this problem is planned.

In conclusion, the unexpected finding that sheepdogs and dogs from households that owned donkeys appeared to have lower odds of coproantigen positivity may be suggestive of a spatial component to transmission in these communities, and will be explored further in future work. A lack of owner knowledge of echinococcosis was found to be associated with higher odds of coproantigen positivity, as was home slaughter. Although it was not possible to distinguish between *E.* *granulosus, E.* *canadensis* and *E.* *multilocularis* infection (all three of which appear to be co-endemic in the study area), these risk factors have previously only been found to be associated with *E.* *granulosus* infection. As well as investigation of potential spatial factors associated with the risk of infection, further work will attempt to identify the species of *Echinococcus* present and evaluate risk factors for these different species. It is also hoped that the results of this and other studies will assist in the development of a comprehensive surveillance strategy including aspects of sampling, coproantigen testing and coproPCR testing, which facilitate the implementation and evaluation of echinococcosis control schemes in Kyrgyzstan and similar areas.

## Acknowledgements

The authors would like to thank Akjol Tagaibekov and Turdumamat Sultanov for fieldwork assistance and hospitality, and Belgees Boufana for laboratory assistance.

## Financial support

This work was supported by the Wellcome Trust (grant number 094325/Z/10/Z).

## Conflict of interest

None.
